# Long waitlists for outpatient drug allergy referrals: An Australian tertiary centre experience

**DOI:** 10.1016/j.waojou.2023.100863

**Published:** 2024-01-09

**Authors:** Syed Ali, Tiffany Hughes, Anthony Smith

**Affiliations:** a Department of Clinical Immunology and Allergy, Flinders Medical Centre, Bedford Park, Australia; b School of Medicine and Public Health, Flinders University, Bedford Park, Australia

**Keywords:** Drug allergy, Drug allergy referrals, Drug allergy waitlist, Outpatient clinic

## Abstract

Drug allergy clinic waitlist time data are limited. A 24-month retrospective study of drug allergy referrals was undertaken at a tertiary hospital in Australia. One hundred six patients were reviewed with a median age of 50 years (IQR 40.5–67.3) and a female predominance (n = 76, 71%). Face-to-face consultations were common (n = 83, 78.3%) with the remainder being telephone consultations.

General practitioners comprised just over one-third (n = 38, 35.9%) of the referrers but majority being from within the hospital, such as the emergency department (n = 22, 20.8%). Most patients (n = 100, 94.3%) were triaged as Category 1 or urgent. Antibiotic allergies were common (n = 75, 70.8%), of which majority were beta-lactam antibiotics (n = 71, 95%): 55 (73.3%) for penicillins and 16 (15.1%) for cephalosporins.

The median waitlist time was 178 days (IQR 48.5–502.5) and only 18 (17%) of Category 1 were seen within urgent timeframe. Telephone consultation had a significantly shorter waitlist time (median 47 days; IQR 6–245) compared to face-to-face consultations (median 267 days; IQR 69–519) (p = 0.026).

Large waitlist times are present for drug allergy, and given the majority of referrers are from hospitals, inpatient drug allergy assessment remains paramount. Beta-lactam antibiotic drug allergy labels remain common, and given their negative implications, further work is needed. Economic and human resources evaluations are required to address this shortfall.

## Introduction

Globally, demand for allergy services continue to grow contributing to long outpatient clinic waitlists. While inherent economical constraints are ever present, in Australia, some limitations are due to most allergy services being provided by specialists. In contrast, some European centres have close involvement of primary care physicians which may improve such outpatient clinic waitlist times.^1^

In South Australia, with a population of 1.8 million inhabitants, the median waitlist time for an Immunology and Allergy specialist outpatient referrals is approximately 540 days, far greater than that of all specialities (177 days).^2^ Data regarding drug allergy clinic waitlist times are limited and formal assessment of drug allergy referral waitlist, particularly in Australia is an important quality improvement exercise. Specifically, earlier evaluation of outpatients with their penicillin allergy referrals is not only associated with a significant reduction in visits, both outpatient and emergency department respectively, but also fewer hospital days.^3^ A retrospective audit was undertaken to establish the outpatient drug allergy waitlist times in a South Australian tertiary hospital.

## Methods

A review of all new patients in the adult Immunology and Allergy clinic was undertaken over a 24-month period (July 31, 2021 to July 31, 2023). Only patients with drug allergy assessment as the primary reason for referral were included in the study.

Electronic medical records were used to obtain data: patient demographics (post code, comorbidities: atopy, metabolic syndrome, and active malignancy), mode of clinic review (telephone or face-to-face consultation), requesting clinician specialty (general practitioner, emergency department, anaesthetists, general medical/medical specialties, and general surgical/surgical specialties), type of drug, and triage category. Triage categories were as follows: Category 1/Urgent (within 30 days), Category 2/Semi-urgent (30–90 days) and Category 3/Non-urgent (90–365 days). Triage categories were assigned at the discretion of the immunologist, however in general Category 1 was reserved for those referrals which outlined concurrent infection requiring antibiotic with the allergy label, recurrent infections in those with antibiotic allergy labels, and drug allergy labels in specific populations (elderly, active solid organ, or haematological malignancies). Waitlist times (days) were calculated by dates of referral to outpatient clinic review. Outcomes of patients who subsequently underwent testing were also recorded.

Descriptive and statistical analyses were undertaken using Graph Pad Prism Version 9.5.1. The study was registered with the Southern Adelaide Local Health Network Quality Register (reference number 4720).

## Results

Five hundred nine new referrals were reviewed over 24 months, of which 106 (20.8%) were specifically for drug allergy. Of these, 23 (21.7%) were via telephone consultation while the remainder were face-to-face consultations.

The median age was 50 years (IQR 40.5–67.3) with a greater proportion of females (n = 76, 71%). Most patients (n = 75, 70.8%) resided in metropolitan regions, while the remainder were in rural regions. Comorbidities in the cohort included atopy (n = 43, 40.6%), metabolic syndrome (n = 27, 25.5%) and concurrent active malignancy (n = 11, 10.4%).

General practitioners (n = 38, 35.9%) and emergency medicine clinicians (n = 22, 20.8%) were the most frequent referrers, followed by medical specialties (n = 16, 15%), general medicine (n = 14, 13.2%), anaesthetists (n = 9, 8.5%), and surgical specialties (n = 7, 6.6%).

Most referrals (n = 100, 94.3%) were triage Category 1, while the remainder were Category 2 (n = 6, 5.7%). Drug classification specific included antibiotics (n = 75, 70.8%), non-steroidal anti-inflammatory drugs (NSAIDs) (n = 12, 11.3%), and radiocontrast media (n = 6, 5.7%). Of the 75 antibiotic specific referrals, the majority were for a beta-lactam antibiotics (n = 71, 95%): 55 (73.3%) for a penicillin-based antibiotic and 16 (15.1%) for a cephalosporin-based antibiotic, respectively.

Collectively, the median time from referral to clinic review was 178 days (IQR 48.5–502.5). Only 18 patients (17%) of Category 1 were seen within the urgent timeframe. Category 1 patients had a significantly shorter waitlist time (median 160 days, IQR 47–446) compared to Category 2 patients (median 573.5 days, IQR506–651.5) (p = 0.001). Patients reviewed via telephone consultation had a significantly shorter waitlist time (median 47 days, IQR 6–245) compared to face-to-face consultations (median 267 days, IQR 69–519) (p = 0.026). There were no significant differences for days on waitlist (days) for general practitioner referrals compared to hospital referrals (p = 0.439) and antibiotic specific allergy referrals compared to other drug allergy referrals (p = 0.363).

There were 3 follow-up subgroups in the cohort: those who had completed testing (n = 63, 59.4%), those who remained on waitlist for testing (n = 23, 21.7%), and those for whom further testing was not required, with formal recommendations made by the treating immunologist (n = 20, 18.9%). Of the 63 patients that had testing, 47 (74.6%) were de-labelled, 13 (20.6%) had confirmation of a specific drug allergy, but showed tolerance to suitable alternatives (eg, cefazolin allergy but tolerance to cephalexin; diclofenac allergy but tolerance to aspirin), and 3 (4.8%) had positive outcomes: 1 with positive intradermal test to trimethoprim/sulfamethoxazole without further challenge, and the other 2 with a positive response to an alternate NSAID, suggesting cyclooxygenase-1 mediated hypersensitivity.

## Discussion

Drug allergy outpatient referrals comprised approximately 20% of all referrals which is higher than that of the literature, around 10%, possibly attributed to geographical and/or ethnic variations, as well as scope of practice amongst specialists. In Australia, specialists in the field have both Immunology and Allergy interests, and therefore may have an over representation in those with autoimmunity conditions over allergic diseases.^4^

More than two-thirds of our cohort were referred by hospital clinicians, highlighting that inpatients are not being actively referred for a formal assessment. Over the last 12-months, our department has promoted this practice, including formal presentations. Therefore, barriers are still present which are not being addressed which may reduce burden for general practitioners. Such clinicians comprised 35% of all referrers in our cohort, which is higher than that reported by others. For example, in a large study of 21 918 patients, only 10% were referred from a primary care provider to specialist for assessment.^4^ Therefore, ongoing education and importance of such a referral needs to be highlighted given the well-established negative implications of an allergy label, particularly for penicillin-based antibiotics.^3^

The long waitlist times at 178 days identified in our cohort for drug allergy patients in tertiary hospital have been highlighted in this study, but is still lower than the specialties overall median wait time of 420 days.^2^ In contrast these waitlist times are long compared to others between 39 and 84 days respectively, with recent United Kingdom targets recommending patients to be seen within 126 days.^5^^,^^6^

Despite high urgency of our outpatient referrals, only 18% were seen within the target 30-day timeframe. Therefore, additional resources are urgently required to target these long delays. For the duration of the retrospective review, the staffing model comprised of 2 part-time immunologists, 1 part-time advanced trainee in Clinical Immunology and Allergy (registrar), and 1 part-time junior medical officer. Given the broad clinical scope of referrals, drug allergy appointments were designated specifically once a week, in a half day clinic by both immunologists and registrar. This clinic was also managing venom referrals, thereby limiting the number of drug allergy referrals that could be seen. Limitations to incorporate further clinic sessions, include lack of funding of additional immunologists and clinic space. To our knowledge, there are no national or international standards for clinical immunologists and allergists. This is likely confounded in Australia, as clinicians do both immunology and allergy, while in some centres around the world, specialists can do stand-alone subspecialty allergy training.

Previously, others have recommended the inclusion of an allergy service run by trained general practitioners or non-clinician practitioners such as pharmacists as well as general increase in clinic availability and staffing.^7^ Additional funding is beneficial, as increasing capacity or engagement of hospital specialists to visit general practitioners has resulted in referral reduction by 39% thereby reducing burden to overall hospital outpatient visits.^8^^,^^9^

Limitations of our study include retrospective nature and relatively small numbers in our cohort. Ideally, a drug allergy specific outpatient clinic should be implemented. Given a large proportion of patients have beta-lactam antibiotics, this may be a specific focus as this inappropriate allergy label has significant negative implications. As patients with telephone consultation in our cohort had a significantly lower waitlist time, implementation of this mode of clinical review can be considered. This may include triage of patients directly to de-labelling, home challenge or further waitlisting for an observed in-hospital challenge.

## Conclusion

Our study has demonstrated that drug allergy referrals are common in the Immunology and Allergy outpatient clinics. A large proportion of patients are not seen within the triage category. Economic and human resources are required to address this shortfall. Larger prospective studies are required in this growing area.

## Abbreviations

IQR, interquartile range; NSAID, non-steroidal anti-inflammatory drug.

## Funding information

The authors received no funding for this study.

## Availability of data and materials

All de-identified data and materials can be provided upon request.

## Author contribution

SA wrote and developed concept for the manuscript. TH, and AS provided to feedback and revisions to manuscript.

## Ethics, consent and permissions

- The study was registered as a quality improvement project with the Southern Adelaide Local Health Network Quality Register (reference number 4720).

- Given the retrospective nature of the study with low to negligible risk, patient consent was not undertaken.

## Consent to publish

- Given no patient identifiers and manuscript focus on waitlist times, no publication consent was obtained from patients.

- All authors have consented for publication.

## Acknowledgements

We would like to acknowledge members of the Immunology and Allergy department at Flinders Medical Centre.

## Declaration of competing interest

All authors have no disclosures to declare.
